# Examining the Role of Testosterone in Mediating Short-Term Aggressive Responses to Social Stimuli in a Lizard

**DOI:** 10.1371/journal.pone.0125015

**Published:** 2015-04-23

**Authors:** Jo McEvoy, Geoffrey M. While, Susan M. Jones, Erik Wapstra

**Affiliations:** 1 School of Biological Sciences, University of Tasmania, Hobart, Tasmania, Australia; 2 Edward Grey Institute, Department of Zoology, University of Oxford, Oxford, United Kingdom; University of Medicine & Dentistry of NJ - New Jersey Medical School, UNITED STATES

## Abstract

Hormones have been suggested as a key proximate mechanism that organize and maintain consistent individual differences in behavioural traits such as aggression. The steroid hormone testosterone in particular has an important activational role in mediating short-term aggressive responses to social and environmental stimuli within many vertebrate systems. We conducted two complementary experiments designed to investigate the activational relationship between testosterone and aggression in male *Egernia whitii*, a social lizard species. First, we investigated whether a conspecific aggressive challenge induced a testosterone response and second, we artificially manipulated testosterone concentrations to examine whether this changed aggression levels. We found that at the mean level, plasma T concentration did not appear to be influenced by an aggression challenge. However, there was a slight indication that receiving a challenge may influence intra-individual consistency of plasma T concentrations, with individuals not receiving an aggression challenge maintaining consistency in their circulating testosterone concentrations, while those individuals that received a challenge did not. Manipulating circulating testosterone concentrations had no influence on either mean-level or individual-level aggression. Combined with our previous work, our study adds increasing evidence that the relationship between testosterone and aggression is not straightforward, and promotes the investigation of alternative hormonal pathways and differences in neuro-synthesis and neuroendocrine pathways to account for species variable testosterone - aggression links.

## Introduction

In the past decade or so, researchers have demonstrated that consistent intra-individual differences in behavioural traits such as aggression are ubiquitous throughout the animal kingdom [1, 2, 3]. However, despite increased attention in this topic, the proximate mechanisms underlying consistent individual differences in behavioural traits remain unclear [4, 5, 6]. Establishing a proximate basis for these traits is key to our understanding of how consistent individual differences in behaviour are generated and maintained, the extent to which traits are correlated with each other, and the role(s) that consistent individual differences in behaviour plays in ecological and evolutionary processes [2, 6, 7, 8, 9, 10, 11].

Hormones have been proposed as a key proximate physiological mechanism that organize, and maintain, consistent intra-individual differences in behaviour [7, 12, 13, 14]. Specifically, hormones are systemic in nature and can simultaneously affect multiple traits [12]. Thus, they have the potential to integrate behavioural traits either through organizational effects acting early during ontogeny, or through activational effects later in life [6, 7]. Organizational effects of hormones are usually thought to organize brain anatomy and neurochemistry and determine the distribution of hormone receptors, as well as act on other aspects of morphology and physiology, all of which set the stage for later activational effects of hormones on behaviour [6, 7]. Activational effects trigger short term expression of context-specific behaviour patterns (e.g. [6, 7]) and can occur through dynamic feedback loops whereby initial social or environmental stimuli elicit an increase (or decrease) in circulating hormone concentrations which, in turn, feedback on a suite of behaviours (e.g. [15, 16]). Both organizational and activational effects represent separate but complementary pathways to the expression of particular behavioural phenotypes and thus may provide a key proximate mechanism that underlies variation in behaviour.

Steroid hormones play a major role in influencing the physiology and behaviour of many animals [17]. For example, the steroid hormone testosterone (T) plays a central role in the regulation of breeding in males as many physiological, morphological and behavioural traits related to reproduction are T dependent [18]). The behavioural trait that is perhaps most commonly linked to T at both an organizational and an activational level is aggression [19, 20]. For example, at an activational level, studies have shown that circulating T concentrations can both respond to, and elicit, aggressive responses to environmental and social stimuli (e.g. [16, 21]). The majority of these studies have suggested a positive activational relationship between T and aggression (e.g., T up-regulates aggression or vice versa). However, recent research has suggested that this is not always the case [6, 15, 22, 23]. Indeed, the relationship between T and aggression can be highly context dependent. Evidence for this comes from work which has shown that high levels of aggression can persist even when T concentrations are very low (i.e. no link between T and aggression), that aggression and T can be negatively correlated, or that other hormones such as progesterone and corticosterone may influence aggressive behaviours [6, 15, 22, 23]. Given that aggression can have a strong influence on the ecological and evolutionary trajectory of populations (e.g. [24, 25, 26]), understanding the ways in which hormones (in particular T) underpin aggression is crucial.

The aim of this study is to investigate the activational relationship between T and aggression in a social lizard species, *Egernia whitii*. Previously, we have demonstrated that free-living male *E*. *whitii* display consistent individual differences in aggression [10, 27]. We have also demonstrated that male *E*. *whitii* display consistent inter-individual differences in circulating baseline T concentrations, and that these baseline T concentrations are negatively linked to a males aggressive phenotype [10]. However, correlations between baseline hormonal concentrations and aggression can be relatively poor indicators of the functional links between hormones and behaviour [28, 29]. Rather, an individual’s ability to produce short-term increases in plasma T may be more important for understanding hormone mediated trade-offs, such as those related to aggression [28]. For example, while T may mediate short-term aggressive responses to social and environmental stimuli, and thus be up-regulated immediately after an aggressive interaction, at the baseline level it may show no overall increase (but instead decrease) with an individual’s aggression phenotype (see [30]). This may be particularly pertinent when both high levels of aggression and high concentrations of T are costly [31]; an activational relationship between the two that allows short-term responses to social and environmental stimuli may therefore enable individuals to limit the costs associated with each [31, 32].

To investigate the activational relationship between T and aggression in male *E*. *whitii*, we tested two complementary hypotheses regarding the functional links between aggression and T. First, we examined whether a behavioural stimulus (a conspecific aggression challenge) caused a change in circulating plasma T concentrations (e.g., the “challenge hypothesis”; [20]) and secondly we examined whether a change in circulating plasma T concentrations caused a change in an individual’s aggressive response to the conspecific challenge. These hypotheses represent contrasting (but complementary) predictions regarding the dynamic interaction between T, aggression and the social environment. Indeed, determining the extent to which activational hormone effects represent a causal or responsive relationship to changes in behaviour is key to understanding these feedback loops and ultimately the role of the endocrine systems as a mediator of behaviour [29, 33].

## Methods

### Study Species

White’s skink (*Egernia whitii*) is a medium sized (up to 100mm snout-vent length, SVL) viviparous skink found throughout grasslands, coastal heath and forests in south-eastern Australia. Individuals are found in discrete patches of open grassland in close proximity to excavated burrows or rock crevices that they use as retreat sites. *Egernia* have an overall lifespan of approximately ten years and reach sexual maturity to approximately three years of age (70 mm SVL) [34]. Tasmanian populations of *E*. *whitii* live in small family groups based on socially monogamous male/female pair bonds, with stable home ranges and approximately 25% extra pair paternity [35]. We used *E*. *whitii* from a population on the east coast of Tasmania, Australia (42°57′ S, 147°88′ E). Individuals in this population are part of a long-term life history study (see [10, 27, 35, 36, 37, 38, 39, 40, 41]. Previous research demonstrates that in male *E*. *whitii* body size is not related to either aggression [10, 27] or plasma T concentrations (10).

#### Ethics Statement

All research was carried out in strict accordance with the Australian Code of Practice for the Care and Use of Animals for Scientific Purposes, 7th edition, 2004 and the University of Tasmanian Animal Ethics Guidelines. All work was carried out under the approval of the University of Tasmania Animal Ethics Committee (Permit Number: A0010061) and the Tasmanian Department of Primary Industries, Parks, Water and the Environment (Permit Number: FA 08245). All individuals from both experiment 1 and experiment 2 were released at their exact point of capture. All lizards were released two days after the conclusion of the experiment to ensure there were no adverse effects from blood sampling, of which there were none.

### Experiment 1: The role of behaviour in activating a T response

Thirty-six adult (>70mm SVL) male *E*. *whitii* were captured in the field on September 25 2012 and immediately sampled (within 2 minutes) for ~ 100 μl blood by venipuncture of the *sinus angularis* (the corner of the mouth) to establish baseline T concentrations. Subsequent to sampling, blood was immediately placed on ice until centrifugation (6000 rpm for 5 min) at the end of the field day. Plasma was stored at −25°C until assayed (see below). Following capture, all individuals were transported back to the specifically designed Terrestrial Ecology facility at the University of Tasmania. Lizards were housed individually in plastic terraria with opaque sides (which allowed for testing only one lizard at a time) in a room maintained at an ambient temperature of 16°C. Each terrarium had a basking light on an 8:16 hr light/dark cycle, and overhead lights on a 9:15hr light/dark cycle. Each terraria had a basking rock under the light at one end, and a shelter at the opposite end (maintained at 15 cm from the closest edge of the basking rock) with water and food (mealworms (*Tenebrio* larvae) or pureed fruit with protein powder) available *ad libitum*. The following day half of the lizards (N = 18) were subjected to a conspecific aggression challenge while the remaining 18 control lizards were not. The aggression challenge consisted of introducing a clay model of a conspecific into the lizards’ home cage to mimic a territorial intrusion and recording the focal lizards’ response (see below for detailed description of our conspecific aggression test protocol). Following the aggression challenge we took a second blood sample from 30 individuals using the same procedure as in the field. Final sample sizes for both pre- and post-aggression challenge samples are N = 16 for the aggression challenge group and N = 14 for the control group: we were unable to obtain sufficient blood to run T assays for the remaining six individuals. As T can rise rapidly following aggressive encounters [31, 42], blood samples were collected 15 minutes after the aggression challenge.

### Experiment 2: The role of T in activating an aggressive response

We captured 95 adult male (> 70mm SVL) *Egernia whitii* between the October 5–21 2011. Following capture, individuals were transported back to the Terrestrial Ecology facilities at the University of Tasmania (see above for animal husbandry). Lizards were then subjected to a conspecific aggression test to establish baseline aggression phenotypes (see below). Individuals were then randomly split into one of four treatment groups (N = 20 for each group): control, sham-control, T-increase and T-block. In the afternoon of the third day, individuals were injected intraperitoneally according to treatment. Treatments were as follows:

1. **Testosterone increase.** T powder (Steraloids, Wilton, NH) was dissolved in sesame oil (18 mg T powder in 20 ml oil with sonication used to fully dissolve the steroid) to make a 0.9 mg/ml final stock solution (see [43] for similar method). This was further diluted 1:2 in sesame oil and injected at a dose of 45 μg T/100 μl sesame oil (see [43] for similar method).
2. **Testosterone block treatment.** we used a combination of the anti-androgen Flutamide, and the aromatase inhibitor 1, 4, 6-androstatriene-3, 17-dione (ATD) (Steraloids, Wilton, NH) dissolved in sesame oil, and injected 18 mg/ml ATD + 14.4 mg/ml Flutamide/100 μl sesame oil (see [43, 44]). This treatment method combines both an aromatase inhibitor (a, 4, 6-androstatriene-3, 17-dione, ATD) and an aromatase antagonist (Flutamide, F) which simultaneously blocks androgen receptors and conversion of T into estradiol, effectively lowering T [31, 43, 44]. As a result Soma et al., [44] found that ATD+F increased plasma T concentrations and suggest that this increase occurred due to negative feedback on luteinizing hormone (LH) secretion being blocked (see also [45]). This elevated plasma T concentration should still have no androgenic effects and thus no effective influence on behaviour as testosterone is blocked from androgen receptors by flutamide (e.g. [44, 45]). Based on this, we would expect either no change or an increase in T concentrations following the block treatment.
3. **Sham-control treatment.** individuals were injected with 100 μl sesame oil.
4. **Control treatment.** these individuals were untreated (no injection).

The following afternoon, individuals received a second (post-treatment) aggression test. Blood samples were analyzed using the T extraction and radioimmunoassay detailed below.

To confirm the validity of the treatment groups, we used the remaining 15 lizards and divided them into a sham-control group, a T-increase or a T-block group (N = 5 per group). These individuals were treated as the experimental lizards were, with the addition of collecting a baseline blood sample on the afternoon pre-treatment and the afternoon post-treatment (at the same time that we conducted conspecific aggression tests on the actual experimental group).

### Conspecific Aggression Test

For both experiment 1 (Aggression Challenge) and experiment 2 (Testosterone Manipulation) we quantified individual aggression with a previously established conspecific aggression test [10, 27, 36, 37, 41, 46]. Aggression tests were conducted in the afternoon between 1300 and 1700 so that lizards could obtain their preferred body temperature before trials began [47]; the test order of individual lizards was randomized on each day.

Conspecific aggression tests consisted of the experimenter (JM) approaching the front of the basking container and touching the lizard with a realistic soft plasticine model of an *E*. *whitii* attached to a wooden dowel. Lizards were presented with the model after a 60 second acclimation period to the presence of the observer, but only if they were found and remained on the basking rock at the start of the test. Models were then advanced to the lizard (snout to snout, as we have observed individuals approaching each other in the wild) and models were presented to the lizard until it fled to the shelter, or up to 10 times in quick succession, with the lizards’ response to the model recorded after each approach. A single trial therefore consisted of up to 10 touches within approximately three minutes (depending on the lizards’ response to the model). We used an act-frequency approach to measuring behaviours in aggression tests [48]. Four behaviours were measured: number of touches before the lizard fled (to a maximum of 10 touches); number of back arches (a display whereby the spine of the lizard was bent to form a concave arch); the number of times the lizard displayed with an open mouth; and the number of times the subject actively bit the model. These aggressive behaviours are identical to those recorded in antagonistic interactions within this and other *Egernia* species (e.g. [27, 49, 50]) and have been observed in aggressive interactions between individuals both in the laboratory and in the wild by both JM and GW. Behaviours in tests were recorded for the entire duration of stimulus presentation with an audiocassette recorder and hand held timer. Behaviours were scored as a multiple frequency if the lizard performed that behaviour anew after each touch with the model.

As with our previous work (see [10, 27, 36, 37, 41, 46]), the four behaviours were highly inter-correlated, loaded strongly on a single common component in Principal Components Analysis (PCA) for each aggression test and explained >67% of the variation at each time point. The Kaiser-Meyer-Olkin (KMO) measure of sampling adequacy and the Bartlett’s test of sphericity both indicated that all matrices were suitable for PCA [51]. We computed aggregate unit-weighted scale scores for each individual per test, resulting in one aggression score (for half of the lizards) in the Aggression Challenge experiment, and two aggression scores for the T Manipulation experiment: one baseline aggression test (pre-treatment), and one post-treatment aggression test.

### Testosterone extraction and Radioimmunoassay (RIA)

Testosterone was measured in extracts of plasma samples by radioimmunoassay following the method described in [52]. Where possible, 25 μl aliquots of plasma were extracted in 500 μl ethyl acetate, although some samples were smaller. Extraction efficiency was 82% and assay results were adjusted accordingly and for sample volume. Duplicate standards (3.25–800 pg authentic T), or 400 μl of plasma extract, were dried down with approximately 5000 cpm tritiated T (Perkin Elmer NET370250UC). The antiserum was Sirosera C-6050, used at a final dilution of 1:30 000 in phosgel (phosphate buffer, pH7.6, with 1% gelatin). The sensitivity of the assay was 3 pg T (≈ 0.1ng/ml plasma). Assay accuracy and precision were assured by including replicates of three levels of commercially available human serum controls (CON6 DPC) plus replicates of two pools of *E*. *whitii* plasma in every assay.

As the lizards are relatively small, we were limited in the amount of blood we could safely take from each individual, especially in the case of repeated sampling. Therefore, we were limited in the extent to which we could measure other hormones, outside testosterone, that could affect aggression or that can have synergistic and agonistic interactions with testosterone, such as estrogen and corticosterone.

### Statistical Analysis

All analysis was conducted using R version 3.0.3 [53]. We were interested in the extent to which our experimental protocols (aggression challenge or T manipulation) influenced both mean concentrations/levels (of T and aggression respectively) and intra-individual consistency (of T and aggression respectively). We thus conducted both mean-level and individual-level analysis. Means ± 1 SE are reported throughout, Intra- Class Correlation coefficient (ICC) is reported with p values and confidence intervals (Upper and Lower confidence intervals: UCI and LCI) for the normally distributed data and with confidence intervals only for the non-normal data (see below).

### Experiment 1: The role of behaviour in activating a testosterone response

Testosterone concentrations were log transformed, and we then analysed the data in two ways. First, we split the data into two data sets, ‘challenge’ and ‘no challenge’. We conducted two linear mixed effects models to examine differences in plasma T at the two time points (pre- and post- aggression challenge, or time one and time two) using the ‘nlme’ package in R [54]. In each case, testosterone concentration was included as the dependent variable, time (one and two, pre and post challenge) as the predictor variable, and ID was included as a random effect to account for the repeated measures nature. A fully factorial mixed model was not possible because our treatment effect (behavioural challenge) is unbalanced—there are three instances where individuals are sampled having not undertaken the treatment (two pre-treatment and one post treatment) and only one where they have undertaken the treatment. To check for differences in the response to the behavioural challenge, we ran a second analysis whereby we calculated the change in plasma T concentrations (delta T) from pre- to post- challenge periods and conducted a one way anova with delta T as the dependent variable and aggression challenge (yes or no) as the independent variable. This allowed us to determine if receiving an aggression challenge influenced the change in plasma T from time one (pre-challenge) to time two (post-challenge). To examine intra-individual consistency in circulating plasma T concentrations we extracted the intra-class correlation coefficient based on an ANOVA model using the rptR package [55]. We first examined overall intra-individual consistency across both treatment groups, and then to determine whether intra-individual consistency in circulating T concentrations differed between treatment groups we ran the model separately for aggression tested and non-aggression tested individuals. We compared intra-individual consistency between treatment groups by comparing associated confidence intervals, and then confirmed this with a Fisher-Z test to see whether correlation coefficients differed between the groups.

### Experiment 2: The role of testosterone in activating an aggressive response

In order to test our treatment protocols, we ran paired t-tests between pre-treatment and post-treatment testosterone concentrations in our additional experimental lizards (see methods, experiment 2). There was no statistically significant increase in T concentrations from pre to post treatment in the sham-control group, however, there was a (non-significant) trend in the sham control towards an increase in T levels from pre- to post- treatment (mean T concentration pre-treatment 3.74 ng/ml ± 1.65 ng/ml, post-treatment 8.30 ng/ml ± 1.88 ng/ml; t_(4)_ = -1.42, p = 0.22) but a five-fold increase in the T-increase group (pre-treatment 3.08 ng/ml ± 0.71 ng/ml, post-treatment 25.02 ng/ml ± 6.64 ng/ml; t_(4)_ = -3.27, p = 0.03). The post-treatment T-increase group concentrations were also significantly higher than the post-treatment sham-control group T concentrations (t_(8)_ = -2.42, p = 0.04) We therefore achieved our intended result of raising T in the T-increase treatment group to within physiological limits experienced by males during the mating season [10]. We also found a three-fold increase in T in the T-block group (pre-treatment 4.24 ng/ml ± 0.56 ng/ml, post-treatment 24.48 ng/ml ± 8.21 ng/ml), however, this increase was not statistically significant (t _(4)_ = -2.55, p = 0.06). Furthermore, post-treatment T-block group concentrations did not differ significantly from post-treatment sham-control group concentrations (t_(8)_ = -1.919, p = 0.09). Given the equivocal effect (a trend but not significant), we ran analysis both with and without this treatment group (see below).

Aggression scores pre- and post-treatment in this experiment were non-normally distributed and were not conducive to transformations. We thus conducted two non-parametric analyses at each of the time points (pre- and post- treatment). Specifically, we used a Kruskal-Wallis test to determine if mean-level pre-treatment aggression scores differed across the groups and we used a second Kruskal-Wallis test to examine whether mean-level post-treatment aggression scores differed across groups. To examine whether T treatment influenced intra-individual consistency in aggression scores we ran a general linear mixed model and extracted the intraclass correlation coefficient using a poisson function [55]. Similarly to the behavioural challenge data, we first examined overall intra-individual consistency (across all treatment groups) and then examined intra-individual consistency in aggression (pre-treatment aggression scores to post-treatment aggression scores) for each treatment group. We compared intra-individual consistency between treatment groups by comparing the confidence intervals, and then conducted a Fisher-Z to confirm differences.

## Results

### Experiment 1: The role of behaviour in activating a testosterone response

**Fig 1:**
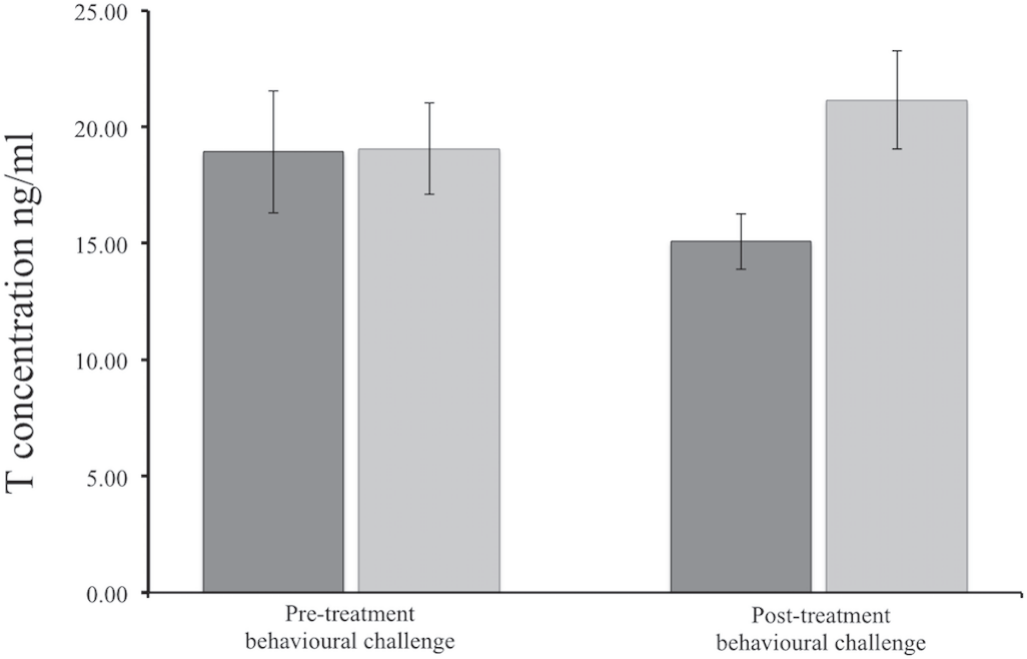
Mean circulating T concentrations (values stated on graph with associated standard error) of male *Egernia whitii* pre- and post- treatment behavioural challenge for those individuals that did not receive a challenge (light grey bars) and those individuals that did receive a challenge (dark grey bars).

Experiencing an aggressive challenge did not appear to influence circulating plasma T concentration at the mean-level, but it did at the individual-level (rank order consistency). Specifically, while Fig 1 suggests that those individuals receiving an aggressive challenge experienced a decrease in circulating plasma T concentrations across time, these effects were not statistically significant (t_(14)_ = -0.58, p = 0.57). We also found that there was no difference in circulating plasma T concentrations across time for those individuals who did not receive the aggression challenge (t_(14)_ = 1.27, p = 0.22). In support of these results we found no difference in the change in plasma T concentrations between those individuals who received the behavioural challenge and those who did not receive the behavioural challenge (F_(1, 28)_ = 1.91, p = 0.17).

**Fig 2:**
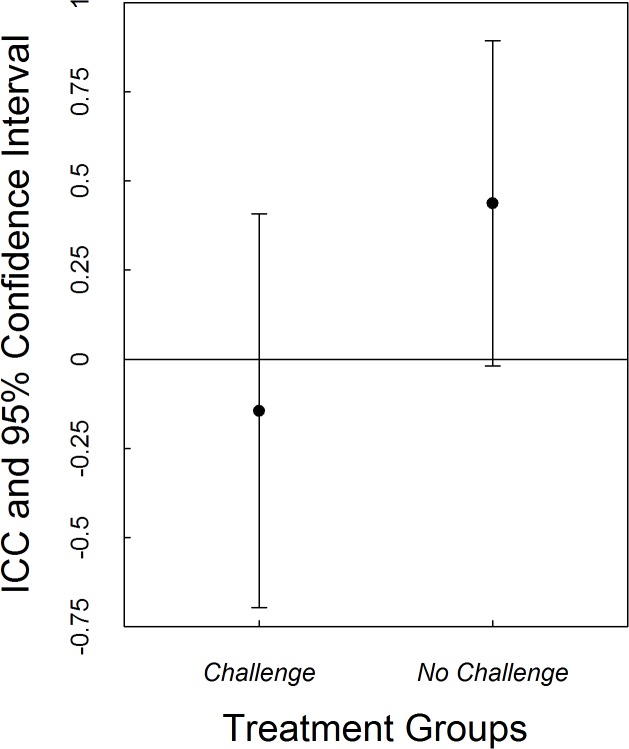
Intra-class correlation coefficients with associated 95% confidence intervals for the consistency of plasma testosterone for those individuals that received a behavioural challenge and those individuals that did not receive a behavioural challenge. Although the ICC values indicate that those individuals not receiving the challenge remained consistent in their circulating plasma T concentrations, the confidence intervals between the two groups overlap, indicating that they are not different from each other.

Overall, individuals were not rank-order consistent in their pre-challenge to post-challenge testosterone concentrations (ICC = 0.157, p = 0.197, LCI = -0.211, UCI = 0.524). However, this appears to be driven by those individuals that received a challenge. Individuals that received the aggression challenge did not exhibit intra-individual consistency in their circulating plasma T concentrations (ICC = -0.145, p = 0.7704, LCI -0.696, UCI 0.407), in contrast, individuals that did not receive the aggression challenge were consistent in their intra-individual T concentrations from pre- to post-challenge (ICC = 0.437, p = 0.0412, LCI -0.019, UCI 0.893). However, variation between individuals in the consistency of their concentrations were relatively high in both groups which resulted in no difference in the consistency between the two treatment groups (Z = -0.861, p = 0.194; see Fig 2).

### Experiment 2: The role of testosterone in activating an aggressive response

There was no difference in mean-level aggression scores pre- or post-treatment across the four treatment groups (pre-treatment aggression across groups: H_(3)_ = 0.326, p = 0.95, post-treatment aggression across groups: H_(3)_ = 2.13, p = 0.54). These non-significant results remained non-significant when we re-ran models excluding the block treatment (because T concentrations in the block treatment were not significantly different from the sham-control group: pre-treatment aggression across groups H_(2)_ = 0.67, p = 0.72; post-treatment aggression across groups H_(2)_ = 0.85, p = 0.65).

**Fig 3:**
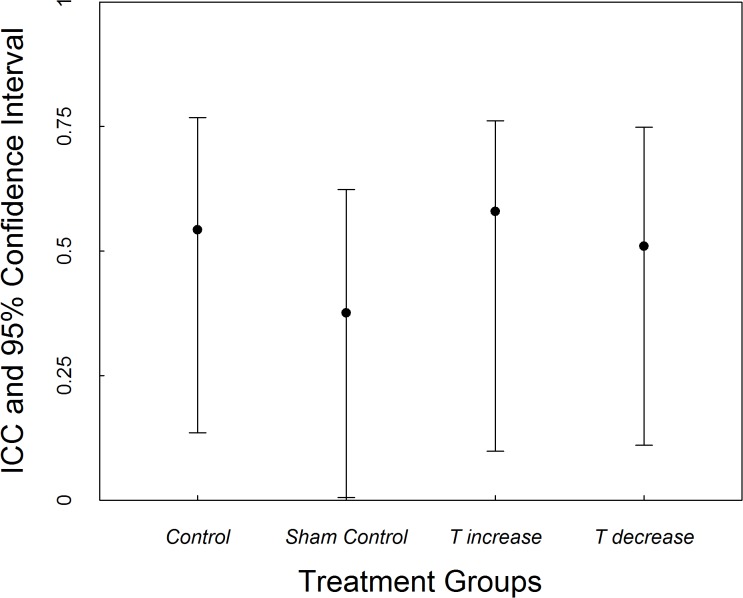
Intra-class correlation coefficients with associated 95% confidence intervals for the consistency of aggression pre- to post- treatment across the four treatment groups. Confidence intervals for all of the groups overlap indicating that the ICC values for each group are not different from each other.

Overall, individuals were consistent in their rank-order aggressive score from pre- to post- treatment (ICC = 0.446, LCI 0.288, UCI 0.598). Consistency estimates varied between the treatment groups (control ICC = 0.543, LCI 0.136, UCI 0.768; sham control ICC = 0.376, LCI 0.011, UCI 0.624; T-increase ICC 0.580, LCI 0.099, UCI 0.764; T-block ICC = 0.510, LCI 0.111, UCI 0.749), but did not differ between treatment groups (no overlap in the confidence intervals: Fig 3). This was confirmed with the Fisher Z statistic testing the difference in the correlation between the smallest and largest ICC value (e.g., between T-increase and sham control; Z = -0.72, p = 0.23).

## Discussion

Hormones commonly integrate distinct behavioural traits either through organizational effects acting early during ontogeny or through activational effects later in life [6, 7]. Activational hormone effects are predicted to result in short-term expressions of type-specific behaviour patterns in response to environmental or social stimuli. Here we examined the short-term relationship between the steroid hormone T and an individual’s conspecific aggressive response in a social lizard species which lives in a highly saturated environment in which conspecific interactions are frequent [27, 34]. We found that experiencing a conspecific aggression challenge did not influence circulating plasma T concentrations, and manipulating underlying T concentrations did not influence in an individual’s aggressive response. Taken together, these results highlight the complexity of the endocrine control of aggression [6, 15, 22, 23, 56]. Below we examine these results in relation to recent advances in our understanding of the functional links between T and aggression, and discuss the role of hormones as a key proximate mechanism that organize, and maintain, consistent intra-individual differences in behaviour.

Our results suggest a novel relationship between plasma T concentrations and aggressive behaviour. Specifically, an aggressive challenge resulted in no impact on T, as opposed to the more traditional view that aggressive behaviour functions to up-regulate T (e.g., [19, 21, 57, 58]). However, we found a suggestion that plasma T concentrations decreased following an aggressive challenge (Fig 1). Although these results were not statistically significant, they do appear to support our previous work which showed negative link between consistent intra-individual variation in aggression scores and baseline circulating T concentrations during the breeding season in male *E*. *whitii* [10]. Combined, this adds to a growing body of work which suggests the relationship between T and aggression is not as straightforward as previously thought [6, 15, 22, 23, 56]. For example, a number of studies have found that T and aggression are unrelated or, in some cases, negatively related (e.g. [23, 29, 59, 60, 61]). In male European robins (*Erithacus rubecula*), house wrens (*Troglodytes aedon*) and Northern cardinals (*Cardinalis cardinalis*) for example, testosterone was not correlated with aggressive behaviours and did not increase (or decrease) in response to stimulated territorial intrusions [23, 60, 61]. Furthermore, deVries et al [61] found this to be the case even though cardinals had the physiological capacity to increase T.

One potential explanation for these results is that aggression is mediated by alternative hormonal mediators. For example, testosterone has been suggested to act as a pro-hormone that needs to be metabolized to estrogens (via aromatization) or dihydrotestosterone (DHT) and other 5α-reduced androgens (via 5α-reduction) before it can affect behaviour and other processes [62, 63]. For example, in female Galápagos marine iguanas (*Amblyrhynchus cristatus*) Rubenstein and Wikelski [29] found an association between increased aggression, increased estradiol and progesterone concentrations, but decreased T concentrations. They suggested that T was rapidly aromatized to estradiol during aggressive encounters, explaining both elevated estradiol and decreased T (see also [62, 63]). It has also been suggested that behaviour may be more influenced by the conversion of androgens to estrogens than by circulating androgens alone [64, 65]. Both estrogen and progesterone have been shown to play a key role in regulating male aggressive behaviour during the non-mating season in various species including lizards (e.g. [57, 58, 59, 60]. Additionally, gonadotrophins have also been shown to have an indirect role in mediating aggressive behaviours [61, 66]) through stimulating the secretion of testosterone (via lutenzing hormone) and gonadotropin-inhibiting hormone has been shown to inhibit aggression in some species [15, 61, 66]. Alternatively, in a number of species it is corticosterone, as opposed to T, that has been shown to respond to territorial challenges [67, 68]. Increases in circulating corticosterone concentrations also serve to suppress circulating T concentrations, thus potentially explaining the negative relationship between T and aggression following an aggressive interaction (e.g. [69, 70]. Differences in neuro-synthesis and neuroendocrine pathways can also account for species variable testosterone—aggression links. For example, it has been suggested that some species may be more sensitive to androgens and utilize lower levels of hormones more efficiently via a more rapid impact of neurosteroids or a greater number of receptors in the brain [56, 61, 71, 72].

These alternative hormonal mechanisms and pathways are suggested to have evolved to decrease the effect of continuously high concentrations of T [31, 59, 61, 71] that would occur if high concentrations of T were linked to high aggressive levels year round. It is possible that a decoupling of the traditional positive relationship between T and aggression has occurred in *E whitii* as adults aggressively compete for and maintain home ranges all year round [38, 39]. Additional work exploring the complicated pathways of steroid metabolism, in which a variety of steroids and their metabolites could be working independently or together to alter aggressive behaviour, is warranted in order to fully elucidate the nature of the feedback loops between social stimuli, aggression and the endocrine system [29, 73].

Social and seasonal factors must also be considered when examining hormonal responses to behavioural challenges. It is possible that dominance rankings, social status, experience and season will also play a role in how individuals respond to behavioural stimulus, including at the physiological level [64, 73, 74, 75, 76]. For example, a number of studies in birds have shown that individuals experiencing a territorial intrusion challenge will have different hormonal responses at different times of the year [61]. This is thought to be a result of individuals experiencing physiological maximums at certain times of the year, during the breeding season for example, and being physiologically unable to respond hormonally during these time periods [61, 64]. Similarly, individuals of differing dominance status may be physiologically constrained in their ability to respond to aggressive encounters. Furthermore, *E*. *whitii* maintain territories year-round, and form largely monogamous pair bonds [39, 40]. Bird species which show no testosterone increase in response to stimulated territorial intrusions possess similar behavioural traits (highly territorial, socially monogamous) [61, 77]. These same traits (long-term territoriality and pair-bonds) may result in stable social situations with little conflict between neighbors whereby transient elevated T to support short-term aggressive interactions is unnecessary. Both DeVries et al [61] and Wikelski et al [77] found evidence that T did not significantly elevate until sometime after exposure to stimulated territorial intrusions, indicating that social instability may influence testosterone-aggression links. It is possible that our experimental design did not capture this possibility. Considering these aspects of the social and demongraphic environment will be an important step in our understanding of the role behaviour plays in mediating hormones and vice versa.

Aggression and testosterone may also be indirectly linked via behavioural syndromes or coping styles. Behavioural syndromes refer to a suite of correlated behaviours [7], while coping style generally tends to refer to a suite of correlated behavioural and physiological traits [13]. This would represent a potentially complicated pathway whereby causing a change in one trait would have indirect flow on effects on another linked trait [7, 13]. Our previous work has shown that aggression is not related to four other major behavioural traits (sociability, activity, exploration and boldness, [41]) indicating a lack of behavioural syndrome and thus aggression is not constrained/influenced by other behavioural traits that may be in turn influence by hormones. The consistency of testosterone that we have observed in this system previously [10], as well as the consistency of aggression that we have seen in this system previously [10, 27, 36, 37, 41, 46] indicates that both of these traits are consistent individual characteristics of *E*. *whitii* which may form part of a hormonal, behavioural, or combined suite of traits in which there are complicated interlinked pathways that may result in apparently unusual or abnormal feedbacks. However, this is unclear at present and deserves further attention.

We also found no concomitant effect of an increase (or decrease) in circulating plasma T concentrations on an individual’s mean aggressive response. These patterns conform to recent work suggesting that an indirect influence of a social stimulus on aggressive behaviour via plasma T is unlikely: instead increased or decreased hormone concentrations should be a consequence of an initial aggressive encounter rather than a cause [33]. Similar results (albeit in the opposite direction) have been reported for a wide number of other species in which an aggressive interaction induces a rapid increase in circulating androgens (including T) (e.g. [33, 74, 75]). This up-regulation has been suggested to function as a facilitator of future competitive interactions [19, 74, 75]. For example, in Californian mice (*Peromyscus californicus*), the effect of a previous win on future contest success is only observed following an increase in circulating T concentrations [16]. In gulf toadfish (*Opsanus beta*), rapid androgen elevation in response to social challenge mediates changes in territorial vocal signaling [78]. In our *Egernia* system, the saturated nature of the habitat means that contests occur frequently. Although we found no effect of manipulating T on aggression, testosterone may function to mediate other aspects of an individual’s behavioural, physical or physiological repertoire in a challenge situation. Further experimental work examining the influence of aggression and testosterone on future competitive interactions is needed in order to tease apart these links.

In terms of individual level consistency (rank order consistency) individuals displayed consistent intra-individual differences in aggression despite the testosterone manipulation, and this fits with all of our previous work [10, 27, 36, 37, 41, 46]. In contrast, we found that there is a suggestion that rank order consistency of circulating plasma T concentrations may be influenced by aggressive experience. We found that individuals who did receive the aggression challenge exhibited a lack of consistency, whereas those that did not receive the aggression challenge exhibited consistency in plasma T concentrations. These results suggest that an aggressive response may influence circulating plasma T concentrations in different ways for different individuals. It has been shown that (similarly) temporal consistency of correlated behavioural traits can be influenced by environmental factors such as predation pressure and social context [7, 13, 14, 79, 80, 81]. The variation in consistency in T between aggressive challenged and non-aggressive challenged individuals observed in this study may provide a mechanistic explanation for these behavioural patterns. Furthermore, when we compare our results from this study with our previous work, it suggests different reactivity of testosterone concentrations over the short and the long term. Specifically, consistency in plasma T concentrations can be variable over the short terms, while long term, baseline plasma T concentrations can be consistent. The variable reactivity of T (temporally, and short term in response to experience) may be beneficial in allowing individuals to cope with challenges, such as frequent conspecific interactions in a saturated environment in the case of *E*. *whitii*, without suffering the potential detrimental effects associated with hormonal changes which may occur with long term changes. We must point out, however, that statistically, these two groups do not differ from each other and thus the conclusions must be interpreted with caution.

What is the relationship between testosterone and aggression in *E*. *whitii*? Complicated. Aggression does not appear to be influenced by testosterone, but circulating plasma T concentration may be influenced by behaviour. There is also a suggestion that the consistency of intra-individual differences in circulating plasma T concentrations may be disrupted by an individual experiencing an aggressive challenge. Overall, these results, combined with our previous work, emphasize the dynamic nature by which the social environment, aggression and T feedback on one another and the potential for responses at multiple levels.

## Supporting Information

S1 Dataset Experiment 1: Refers to the Behavioural Challenge experiment. ‘AggTest’ is the aggression test that was given to half of the individuals, 1 = did receive an aggression test, 2 = no aggression test. ‘Time’ refers to the first or second sample, 1 = is the testosterone concentration sample that was initially taken, 2 = the testosterone concentration sample that was taken after the aggression test. ‘Testos’ is the circulating testosterone concentration in ng/ml. Experiment 2: Refers to the experiment in which we manipulated testosterone concentrations. ‘Treat’ refers to the treatment group, 1 = control, 2 = sham control, 3 = testosterone increase, 4 = testosterone block. ‘Week’ refers to the time points, 1 = week 1 when we conducted pre-treatment aggression tests, 2 = week two when we conducted post-treatment aggression tests. ‘Agg’ refers to the aggression score for each individual. Exp2 treat check: refers to the extra animals that we treated in order to test that the treatments used in experiment 2 worked. Treatment groups are the same as above, and testosterone concentrations were measured pre and post treatment.
(XLSX)
